# Decreased exosome-delivered miR-486-5p is responsible for the peritoneal metastasis of gastric cancer cells by promoting EMT progress

**DOI:** 10.1186/s12957-021-02381-5

**Published:** 2021-10-23

**Authors:** Xian-Ming Lin, Zhi-Jiang Wang, Yu-Xiao Lin, Hao Chen

**Affiliations:** 1 Department of General Surgery, The Second Affiliated Hospital of Zhejiang University School of Medicine, Hangzhou 310009 Zhejiang, People’s Republic of China; 2 Next Generation Sequencing, DIAN Diagnostics Group Co., Ltd., Hangzhou 310030 Zhejiang, People’s Republic of China; 3 Next Generation Sequencing, Hangzhou Dian Huayin Biotechnology Co., Ltd., Hangzhou 310030 Zhejiang, People’s Republic of China; 4 Safety Evaluation Center, Zhejiang Academy of Medical Sciences, Hangzhou 310007 Zhejiang, People’s Republic of China

**Keywords:** Exosome, miR-486-5p, Gastric cancer, Epithelial-mesenchymal transition

## Abstract

**Background:**

The present study aims to investigate the preliminary mechanism underlying the peritoneal metastasis of gastric cancer cells.

**Methods:**

Exosomes from GC9811 cells (Con-Exo) and from GC9811-P cells (PM-Exo) were extracted by ultracentrifugation, which were identified with transmission electron microscopy (TEM) and nanoparticle trafficking analysis, as well as the expression of CD9, CD63, and CD81 detected by Western blot assay. *α*-SMA expression was determined by immunofluorescence assay and Western blot assay. The levels of Snail1, E-cadherin, and Actin-related protein 3 (ACTR3) were evaluated by Western blot assay. MiRNA array was performed on exosomes to screen the differentially expressed miRNAs. The expressions of miRNAs, *SMAD2*, *CDK4*, and *ACTR3* were determined by QRT-PCR. The delivery of miR-486-5p was confirmed by laser confocal detection.

**Results:**

Firstly, TEM, nanoparticle trafficking analysis, and Western blot assays were used to confirm the successful extraction of Con-Exo and PM-Exo. The incubation of Con-Exo and PM-Exo could decrease E-cadherin expression and increase of *α*-SMA respectively in HMrSV5 cells, with the increased proportion of fusiform cells. More significant changes were observed in PM-Exo-treated HMrSV5 cells. Secondary, compared to Con-Exo, miR-486-5p and miR-132-3p were found downregulated, and miR-132-5p was found upregulated in PM-Exo. The transfection of miR-486-5p and miR-132-3p was observed to suppress EMT, and the transfection of miR-132-3p was observed to induce EMT. Laser confocal detection confirmed the delivery of miR-486-5p from gastric cancer cells to HMrSV5 cells through exosomes. Lastly, the expression of Mothers against decapentaplegic homolog 2 (SMAD2), cyclin-dependent kinase 4 (CDK4), and ACTR3 was found to be downregulated via miR-486-5p.

**Conclusion:**

Decreased delivery of miR-486-5p via exosomes might be responsible for the peritoneal metastasis of gastric cancer cells by promoting epithelial-mesenchymal transition progress.

## Introduction

Gastric cancer is commonest malignant tumor of the stomach with relatively high mortality rates globally [1]. In China, gastric cancer is reported with high prevalence, and about 400,000 new patients are diagnosed with gastric cancer annually, which occupies 42% of the total number of patients globally [2]. There are many environment and genetic factors that contribute to increasing a person’s chance of developing gastric cancer, which contains age, gender, bacteria, genetics, ethnicity, diet, health conditions, occupational exposure, tobacco, alcohol, and obesity [3]. Peritoneal membrane is reported to be the commonest site of metastasis and recurrence in patients with gastric cancer [4]. Peritoneal metastases are one of the main elements that result in death. The median survival rate of non-metastasic gastric cancer patients is 14 months, while the median survival rate of patients with peritoneal metastasis is around 4 months [5]. Therefore, it is of great scientific and clinical significance to explore the pathological mechanism of peritoneal metastasis of gastric cancer for the diagnosis and treatment of advanced gastric cancer.

Peritoneal mesothelial cells are the main components of peritoneal tissue, and it is reported that the peritoneal metastasis of gastric cancer is closely related to the interaction between gastric carcinoma cells and peritoneal mesothelial cells. Epithelial-mesenchymal transition (EMT) in the peritoneal mesothelial cells can be induced by TGF-*β* released from gastric carcinoma cells, which further contributes to the processing of fibrosis and the enhancement of cell migration and invasion [6–8]. The peritoneal metastasis of gastric cancer could be suppressed by the inhibited EMT progression by blocking TGF-*β*1 signaling [9, 10]. Therefore, EMT might be an effective target for the prevention of peritoneal metastasis of gastric cancer. However, it is currently unclear that how gastric carcinoma cells regulate the processing of EMT to achieve peritoneal metastasis.

Recently, exosomes are reported to be involved in intercellular communication and play an important role in regulating cellular bio functions and the development of multiple diseases [11–13]. It is currently reported that the progress of EMT in the peritoneal mesothelial cells could be triggered by the exosomes released from gastric carcinoma cells, which finally contributes to the peritoneal metastasis of gastric cancer [14]. Tissue or cell-specific proteins, lipids, and fragments of nucleic acids are abundant in the exosomes, which can be delivered by horizontal transfer or cytokine-cytokine receptor interaction to impact the other cellular functions. For example, exosomes that are rich in wnt3a and released from gastric carcinoma cells can be ingested by the peritoneal mesothelial cells, which results in the infiltration of peritoneal mesothelial cells and the invasion of gastric carcinoma cells [15]. In addition, exosomes isolated from gastric carcinoma cells are rich in miR-106a and are delivered to the peritoneal mesothelial cells by horizontal transfer, which further regulates the apoptotic and migration ability of gastric carcinoma cells by mediating the processing of EMT through targeting Smad7 and finally contributes to the development of peritoneal metastasis [16]. MiRNAs are groups of endogenic non-coding RNAs with regulatory functions produced from series of nuclease shear processing. MiRNA and transcription factors regulation network of hub genes may provide potential prognostic values in gastric cancer [17]. Multiple reports claimed the involvement of miRNAs in the regulatory function of exosomes in the development and processing of malignant tumors [18, 19]. In the present study, the specific miRNA was screened in the exosomes released by peritoneal metastatic gastric cells to explore the potential mechanism of peritoneal metastasis.

## Methods

### Cell lines and treatments

The human non-metastatic gastric cancer cells, GC9811 cells, the subtype of GC9811 cells with high peritoneal metastatic potential, GC9811-P cells, and the human peritoneal mesothelial cell line HMrSV5 cells were obtained from ATCC (Boston, USA). These cells were cultured in the RPMI-1640 medium containing 10% fetal bovine serum (FBS) and at the condition of 37 °C and 5% CO_2_ [20].

### Isolation and purification of exosomes from GC9811 cells (Con-Exo) and from GC9811-P cells (PM-Exo)

The procedures of the isolation and purification of Con-Exo and PM-Exo were conducted according to the instruction described previously [21]. In brief, the GC9811 cells or GC9811-P cells were cultured in the 1640 medium containing 10% Exo-FBS for 48 h, followed centrifuging the supernatant at 3000 rpm for 15 min to remove cells. Subsequently, the samples were filtrated with 0.22 μm filter to remove cell debris, followed by precipitating exosomes using the ExoQuick TC (System Biosciences, California, USA) according to the instruction of the manufacturers. Lastly, the exosomes were resuspended in the PBS buffer and stored at −80 °C for subsequent experiments.

### Transmission electron microscopy (TEM)

The 200-mesh copper electron microscopy grids pre-coated with formvar carbon were added with exosomes pellet, then incubated for 5 min and stained with standard uranyl acetate. The samples were washed three times with PBS buffer and the observed under the transmission electron microscope (Tecnai Spirit TEM T12, FEI, USA) at room temperature following semi-dry.

### Nanoparticle trafficking analysis

The NanoSight NS300 system (NanoSigth, Malvern, UK) was used to detect the absolute size distribution of isolated exosomes for verification. Briefly, the isolated exosomes were diluted with PBS buffer and the particles were automatically tracked and sized based on Brownian motion and the diffusion coefficient. The measurement condition was settled as 23.75 ± 0.5 °C, 25 frames per second, and 60 s measurement time.

### MiRNA microarray of exosomes

The Con-Exo and PM-Exo were collected for microarray analysis using the Agilent Human miRNA microarray (Agilent Technologies, California, USA). Briefly, miRNAs were labeled and hybridized using the miRNA Complete Labeling and Hybridization kit (Agilent Technologies, California, USA) based on the instruction of the manufacturer [22]. The data was extracted using the Feature Extraction software (Agilent Technologies, California, USA), and the Gene Spring GX software 11.0 (Agilent Technologies, California, USA) was used to normalize the signals.

### Real-time PCR assay

After isolating the total RNA from the cells using the TRIzol kit (CWBIO, Shanghai, China), the isolated RNA was further transcribed into cDNAs using the reverse transcription kit (Baosheng, Dalian, China). The relative expression profiles of miRNAs, *SMAD2*, *CDK4*, and *ACTR3* were determined by fluorescence quantitative polymerase chain reaction at 95 °C for 30 s, 55 °C for 40 s, and 72 °C for 45 s according to the instruction described previously with the cDNA template [23]. RUN6-1 (U6) snRNA and Glyceraldehyde-3-Phosphate Dehydrogenase (GAPDH) were utilized for normalization, and the 2^−ΔΔCt^ method was used for quantification.

### Western blot analysis

The proteins were extracted from the exosomes or cells using the lysis buffer, followed by being quantified using the BCA kit (CWBIO, Shanghai, China). Subsequently, approximately 40 μg proteins were loaded and separated using the SDS-PAGE, which were further transferred to the PVDF membrane (Thermo, MA, USA). After washing with tris-buffered saline with 0.1% Tween® 20 detergent (TBST) buffer, the membrane was incubated with 5% bovine serum albumin (BSA) to remove the non-specific binding proteins, followed by being washed and incubated with primary antibodies against CD9 (1:1000, CST, Boston, USA), CD63 (1:1000, CST, Boston, USA), CD81 (1:1000, CST, Boston, USA), E-cadherin (1:1000, CST, Boston, USA), *α*-SMA (1:1000, CST, Boston, USA), Snail (1:1000, CST, Boston, USA), ACTR3 (1:1000, CST, Boston, USA), or GAPDH (1:1000, CST, Boston, USA) at 4 °C overnight. Subsequently, the membrane was washed and incubated with the secondary antibody (1:500, CST, Boston, USA) at room temperature for 1.5 h. Finally, the bands were exposed to Tanon 1600/1600R (Tanon, Shanghai, China) after adding the ECL solution and the images were quantified using the Image J software [24].

### Immunofluorescence staining

The treated HMrSV5 cells were fixed with ice-cold methanol at 4 °C for 10 min, followed by being washed three times using the PBS buffer. Afterwards, the cells were permeabilized in 0.1% Triton X-100/PBS for 10 min at room temperature, followed by removing the non-specific binding proteins using the 5% BSA solution for 30 min. Subsequently, the cells were incubated with primary antibodies against *α*-SMA (CST, Boston, USA) at 4 °C overnight, followed by being incubated with secondary antibody (Invitrogen, California, USA) for 1 h at room temperature. Finally, the cells were washed and stained with Hoechst 33342 (Gibco, New York, USA) to visualize the nuclei [25].

### Laser confocal detection

The treated HMrSV5 cells were seeded on 12-well plates and fixed with 3% formalin at room temperature for 10-15 min, followed by being washed three times. The fixed cells were placed on 12-well plates and perforate using 1% Triton-X 100, which were further incubated at room temperature for 5-10 min. After washing for three times, the cells were incubated with 5% BSA solution at room temperature for 30 min to remove the non-specific binding proteins, followed by being incubated with primary antibody against Cys 3 (1:200, CST, Boston, USA) at 37 °C for 1 h. Following washing with PBS buffer for three times, DAPI was added to be incubated at room temperature for 30 min, followed by 3 times washes and sealing on the glass slides with fluorescence quenched sealing reagents (Invitrogen, California, USA). After fixing the slides with nail enamels, the images were taken using a confocal laser scanning microscope (Olympus, Tokyo, Japan) [26].

### Statistical analysis

SPSS 21.0 statistical software was used to analyze the data, which were expressed as the mean ± standard deviation. Comparisons between the two groups were conducted using *t* test. One-way ANOVA one-way ANOVA assessed comparisons among multiple groups assessed comparisons among multiple groups. *P* < 0.05 was considered to indicate a statistically significant difference.

## Results

### Con-Exo and PM-Exo were successfully extracted and identified

**Fig. 1:**
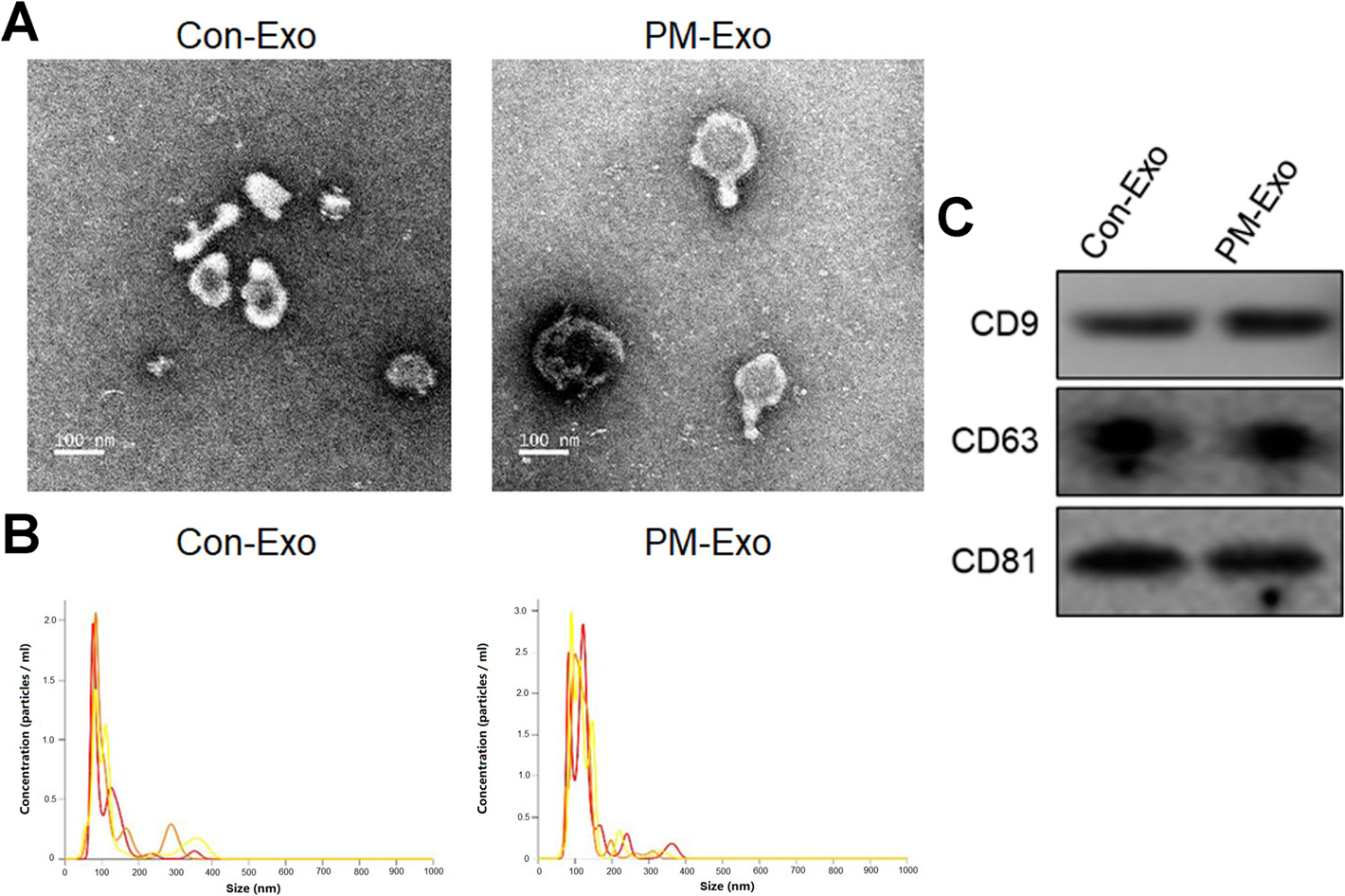
The Con-Exo and PM-Exo were successfully extracted and identified. **A** The morphology of the extracted exosomes was observed using TEM. **B** The median particle size of the extracted exosomes was analyzed by nanoparticle trafficking analysis. **C** The expression level of CD8, CD63, and CD81 in the extracted exosomes was determined by Western blot assay

According to the isolation instruction of exosomes, the Con-Exo and PM-Exo were isolated from GC9811 and GC9811-P cells, respectively. As shown in Fig. 1A-B, the medium particle size of extracted Con-Exo and PM-Exo was around 100 nm, consistent with the medium particle size reported on exosomes [32029601]. In addition, high expression of the biomarker of exosomes, including CD8, CD63, and CD81, was observed in both Con-Exo and PM-Exo. These data indicated that the Con-Exo and PM-Exo were isolated and purified successfully.

### EMT processing in the HMrSV5 cells was promoted more significantly by PM-Exo than Con-Exo

**Fig. 2:**
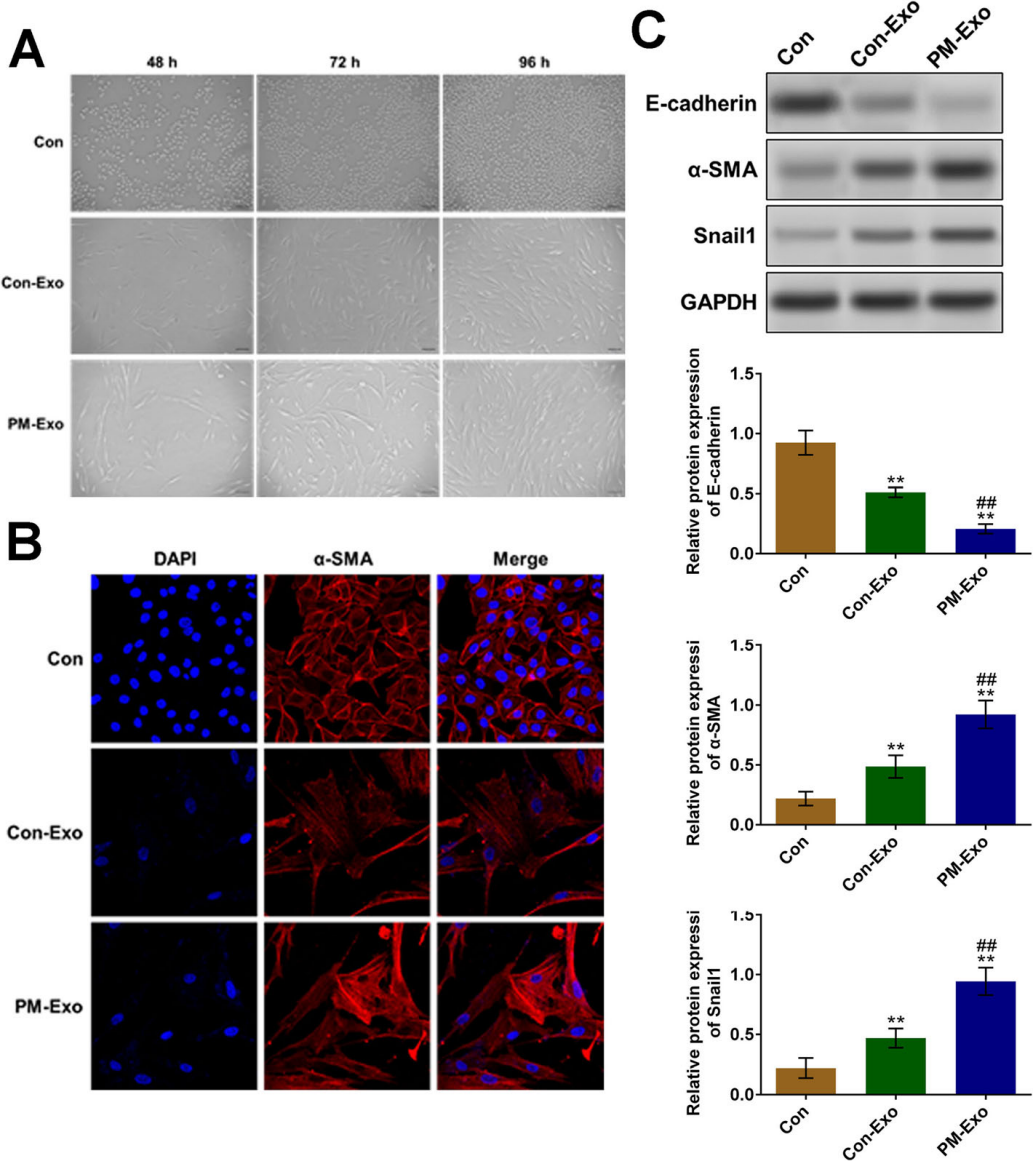
More significant EMT processing was observed in PM-Exo-treated HMrSV5 cells than in Con-Exo-treated HMrSV5 cells. **A** The morphology of treated HMrSV5 cells was observed using the inverted microscopes. **B** The expression of *α*-SMA in treated HMrSV5 cells was determined with immunofluorescence staining assay. **C** The expression of E-cadherin, *α*-SMA, and Snail1 in treated HMrSV5 cells was evaluated by Western blot (**p < 0.01 vs. control, ##*p* < 0.01 vs. Con-Exo)

To evaluate the impact of Con-Exo and PM-Exo on the EMT processing in human peritoneal mesothelial cells, the morphology of treated HMrSV5 cells and the expression of EMT-related proteins were evaluated. As shown in Fig. 2A, compared to control, the morphology of HMrSV5 cells treated with Con-Exo and PM-Exo was significantly altered, with more fusiform cells involved, which was more significant in PM-Exo-treated HMrSV5 cells. According to the images of immunofluorescence staining (Fig. 2B), a significant higher expression of *α*-SMA was observed in PM-Exo-treated HMrSV5 cells, compared to control. As shown in Fig. 2C, the expression of epithelial-related protein, E-cadherin, was significantly suppressed in Con-Exo- and PM-Exo-treated HMrSV5 cells (**p < 0.01 vs. control). In addition, compared to Con-Exo, the expression of E-cadherin in PM-Exo-treated HMrSV5 cells was dramatically inhibited (##*p* < 0.01 vs. Con-Exo). Compared to control, *α*-SMA and Snail1 were pronouncedly upregulated in Con-Exo, and PM-Exo-treated HMrSV5 cells (**p < 0.01 vs. control). Compared to Con-Exo, higher expression of *α*-SMA and Snail1 was observed in PM-Exo-treated HMrSV5 cells (##*p* < 0.01 vs. Con-Exo). These data indicated that EMT progressing was induced in HMrSV5 cells treated with Con-Exo and PM-Exo, which was more significant in PM-Exo-treated HMrSV5 cells.

### MiRNAs array screening on Con-Exo and PM-Exo

**Fig. 3:**
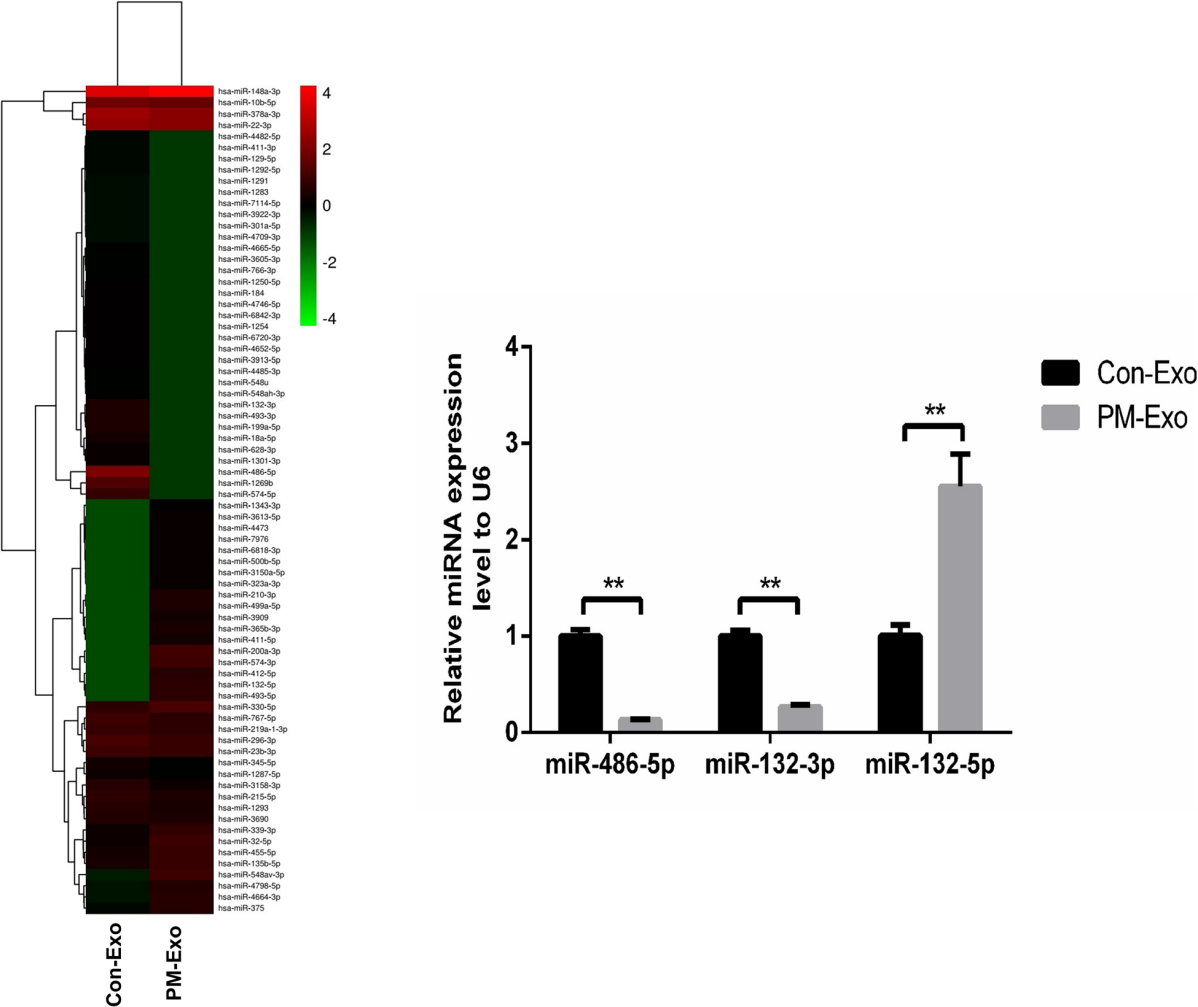
MiRNAs array screening on Con-Exo and PM-Exo. **A** The relative expression level of miRNAs was screened using the Agilent Human miRNA microarray. **B** The expression level of miR-486-5p, miR-132-3p, and miR-132-5p were determined by qRT-PCR assay (**p < 0.01 vs. Con-Exo)

To explore the potential mechanism underlying the regulatory effect of exosomes on EMT, the miRNA assay was performed on Con-Exo and PM-Exo. As shown in Fig. 3A, the thermodynamic diagram of the miRNAs indicated that compared to Con-Exo, miR-486-5p and miR-132-3p were significantly down-regulated in PM-Exo and miR-132-5p was found pronouncedly upregulated in PM-Exo. We further verified the expression level of the three miRNAs in Con-Exo and PM-Exo using qRT-PCR assay. As shown in Fig. 3C, the result of qRT-PCR is consistent with the data obtained from the miRNAs array (**p < 0.01 vs. Con-Exo).

### MiR-486-5p delivered by exosomes was involved in the regulation of EMT

**Fig. 4:**
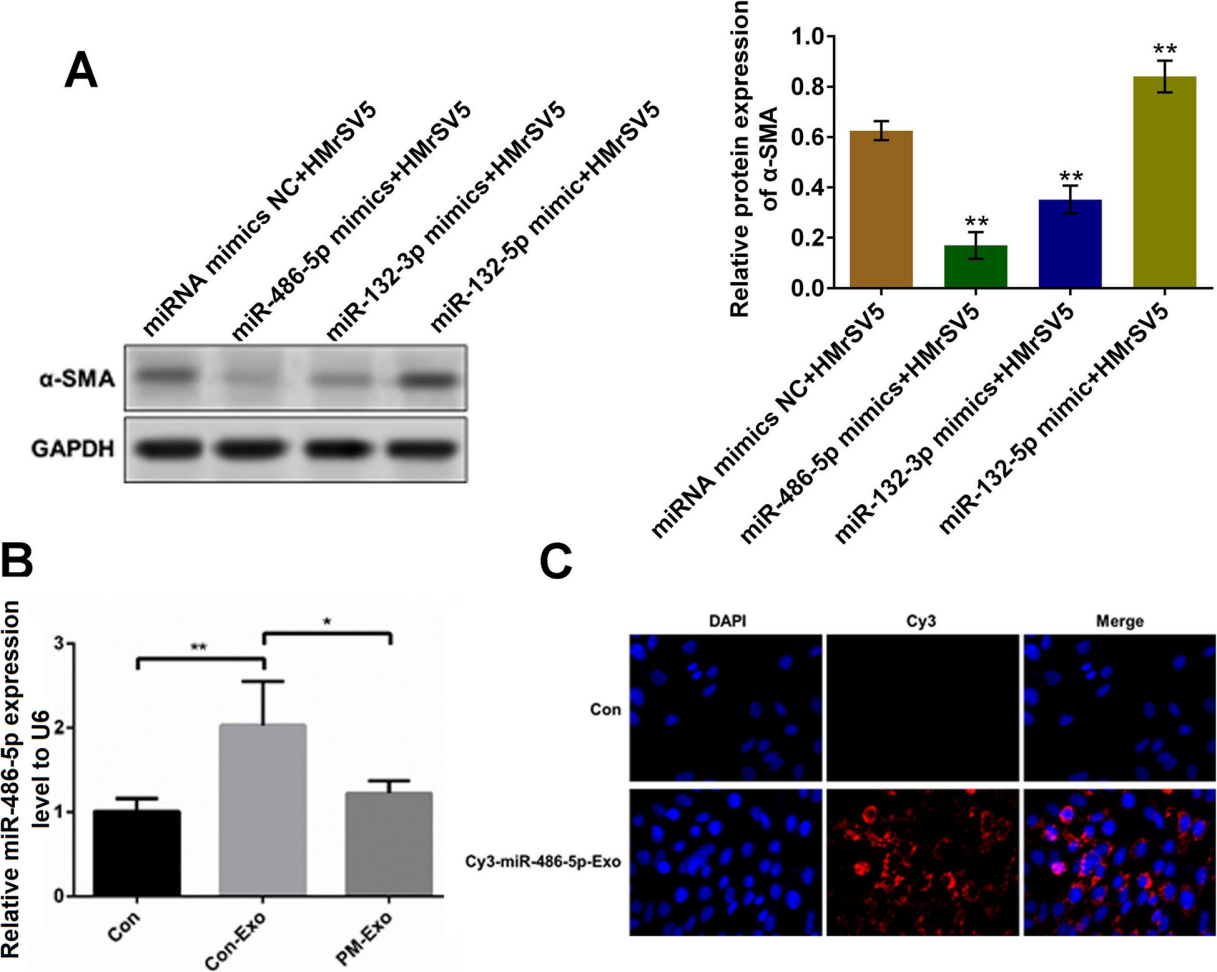
MiR-486-5p delivered by exosomes regulated the processing of EMT. **A** The expression level of *α*-SMA in HMrSV5 cells transfected with different miRNAs was determined by Western blot (**p < 0.01 vs. miRNA mimics NC). **B** The gene expression of miR-486-5p was detected by qRT-PCR (*p < 0.05, **p < 0.01). **C** The expression of miR-486-5p was imaged using laser confocal microscopy

We further transfected HMrSV5 cells with miRNA mimics NC, miR-486-5p mimics, miR-132-3p mimics, and miR-132-5p mimics, respectively. As shown in Fig. 4A, compared to miRNA mimics NC, the expression of *α*-SMA was significantly inhibited by the introduction of miR-486-5p mimics or miR-132-3p mimics and was dramatically elevated by the transfection of miR-132-5p mimics (**p < 0.01 vs. miRNA mimics NC), among which, the expressional difference of *α*-SMA induced by miR-486-5p mimics was the most significant. Afterward, HMrSV5 cells were incubated with blank medium, Con-Exo, and PM-Exo, respectively, and the expression of miR-486-5p was detected. As shown in Fig. 4B, we found that compared to control, the expression of miR-486-5p was significantly promoted in HMrSV5 cells incubated with Con-Exo (**p < 0.01). And compared to Con-Exo, the expression of miR-486-5p was greatly lower in HMrSV5 cells incubated with PM-Exo (*p < 0.05), indicating that the relative expression level of miR-486-5p in HMrSV5 cells incubated with Con-Exo and PM-Exo was consistent with that in Con-Exo and PM-Exo, respectively. To confirm that miR-486-5p was delivered into HMrSV5 cells by the exosomes, we transfected GC9811-P cells with Cys labeled miR-486-5p (Cys-miR-486-5p) and isolated the exosomes, which were further used to incubate with HMrSV5 cells. As shown in Fig. 4C, compared with control, significantly increased red fluorescence was observed in Cys-miR-486-5p-Exo incubated HMrSV5 cells, indicating that miR-486-5p was delivered into HMrSV5 cells by exosomes released by GC9811-P cells.

### The expression of ACTR3 was regulated by miR-486-5p

**Fig. 5:**
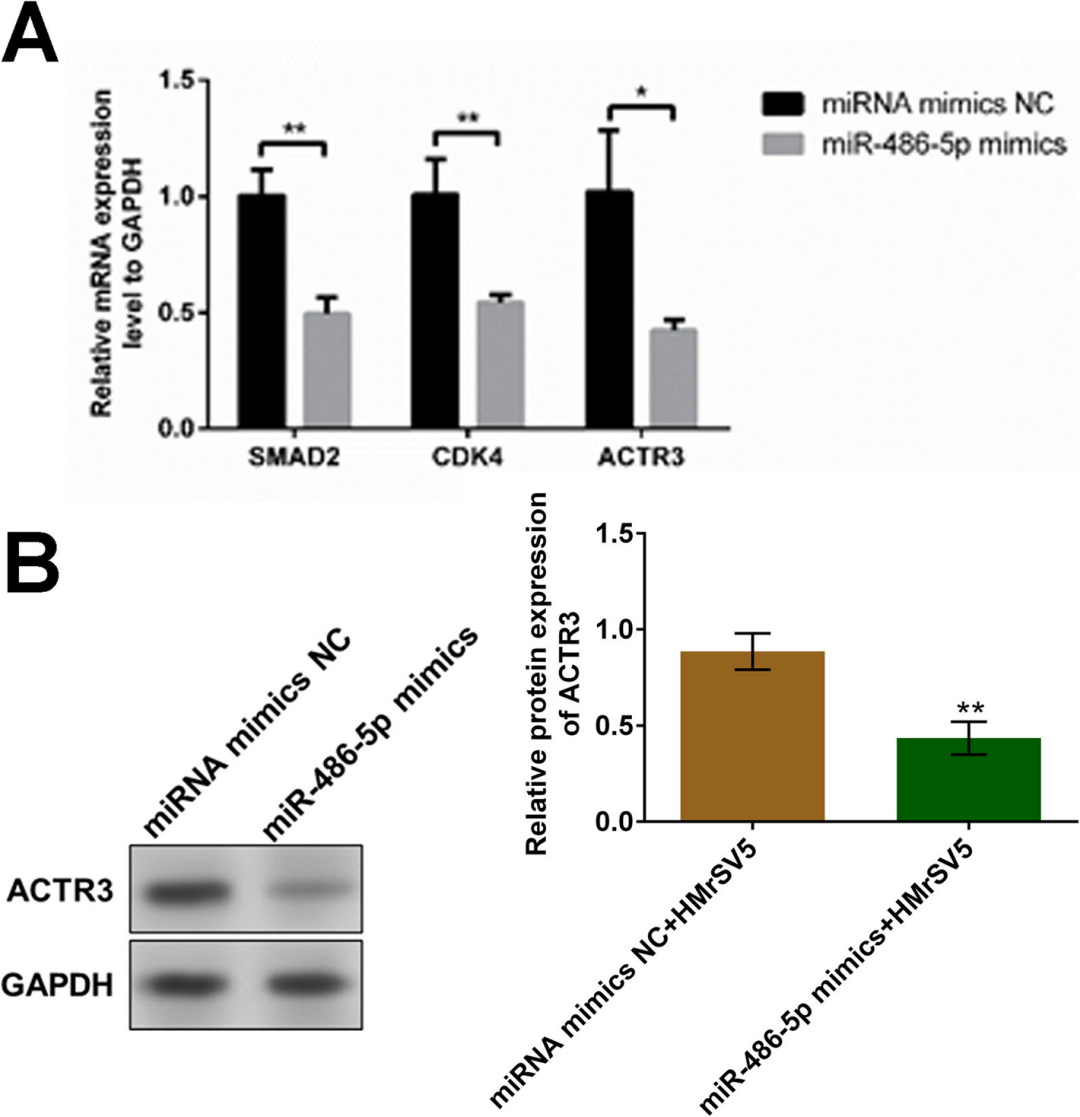
miR-486-5p regulated the expression of ACTR3 in HMrSV5 cells. **A** The gene expression of *SAMD2*, *CDK4*, and *ACTR3* in HMrSV5 cells was determined by qRT-PCR (*p < 0.05, **p < 0.01). **B** The protein expression level of ACTR3 in HMrSV5 cells was evaluated by Western blot assay (**p < 0.01 vs. miRNA mimics NC)

To further explore the potential target of miR-486-5p for the regulatory function against EMT, HMrSV5 cells were transfected with miRNA mimics NC and miR-486-5p mimics, respectively. As shown in Fig. 5A, the gene expression of *SAMD2*, *CDK4*, and *ACTR3* was significantly suppressed by the introduction of miR-486-5p mimics (*p < 0.05, **p < 0.01). In addition, protein ACTR3 was found dramatically downregulated by the transfection of miR-486-5p mimics (**p < 0.01 vs. miRNA mimics NC).

## Discussion

EMT is involved in the proliferation, migration, and invasion of multiple types of malignant tumors, including gastric cancer [27]. When EMT process initiates, the cell junctions in epithelial cells will be disconnected, and the degradation of related ligandins will be induced [28]. In addition, the expression of polar protein complex will also be inhibited, and the morphology of epithelial cells will be changed from classic pebble form into fusiform, which is the morphology of mesenchymal cells. Simultaneously, the cell integrity will be disrupted, the adhesion ability will decline, and the migration ability will be enhanced, which further induces the tumor cells to migrate from primary lesion to the distant area [29]. In the present study, we found that the morphology of HMrSV5 cells treated with Con-Exo and PM-Exo was changed to fusiform obviously, indicating that EMT was initiated. In addition, the ratio of fusiform cells in HMrSV5 cells treated with PM-Exo was higher than that in HMrSV5 cells treated with Con-Exo, indicating that the more significant EMT was induced by PM-Exo than Con-Exo. It is currently reported that the processing of EMT could be reflected by the expression of EMT-related biomarkers [30]. In the progress of EMT, the adhesion connections in epithelial cells are dismissed and its key protein, E-cadherin, is transformed into N-cadherin, which is a biomarker of mesenchymal cells, accompanied by the upregulation of N-cadherin and the downregulation of E-cadherin. In addition, the transformation from E-cadherin to N-cadherin is regulated by related transcriptional factors, such as Snail family and *α*-SMA, which inhibit the expression of E-cadherin and promote the expression of N-cadherin [31]. In the present study, we found that EMT was significantly induced in HMrSV5 cells by the incubation of PM-Exo and Con-Exo, which was verified by the elevated expression level of Snail and *α*-SMA, as well as the decreased expression of E-cadherin. In our future work, the expression of N-cadherin will be detected to further verify the production of mesenchymal cells.

**Fig. 6:**
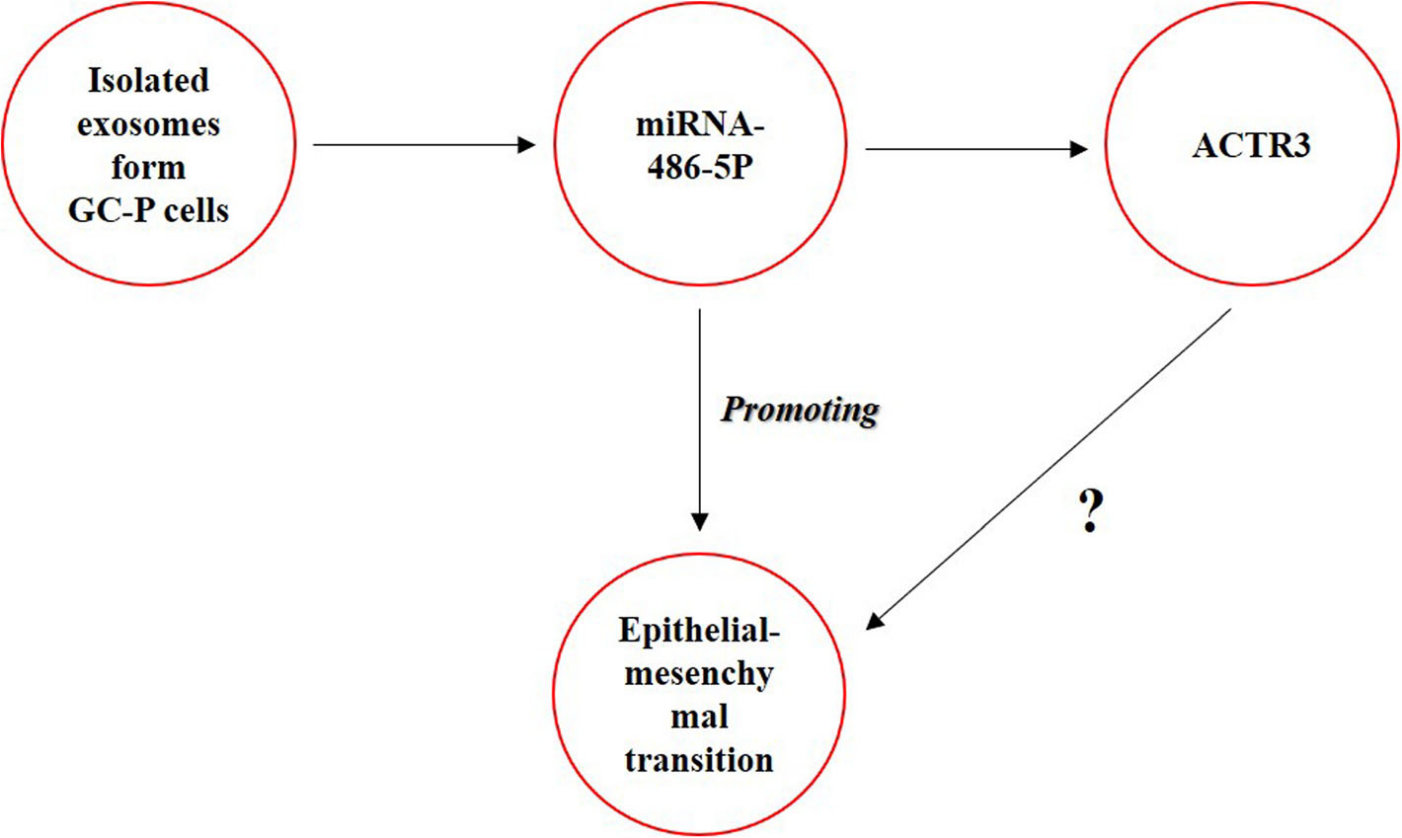
The main pathway of the evaluated factors for peritoneal metastasis in gastric cancer

The regulatory function of miRNAs on EMT processing has been widely reported. MiR-406 upregulates E-cadherin expression by targeting Snail2 to suppress the EMT progress [32]. MiR-204 inhibits the phosphorylation of SMAD2 and SMAD3 by directly targeting TGF-*β* receptor 2, which further contributes to the inhibition of EMT and elevates the sensitivity to 5-fluorouracil in gastric cancer cells [33]. In this study, the differentially expressed miRNAs were screened in PM-Exo and Con-Exo with a miRNA array. We found that miR-486-5p and miR-132-3p were significantly downregulated in PM-Exo, indicating that miR-486-5p and miR-132-3p might be a suppressor of the EMT processing. Then, we further transfected the miR-486-5p mimics and miR-132-3p mimics into HMrSV5 cells, respectively. The change of expression level of *α*-SMA indicated that miR-486-5p and miR-132-3p significantly suppressed EMT. And, the impact of miR-486-5p on the expression level of *α*-SMA was more significant. The regulatory impact of miR-486-5p on EMT in the present study was consistent with the reports claiming the considerable function of miR-486-5p in mediating EMT in d [34–37]. Furthermore, our data revealed that miR-486-5p was delivered into mesothelial cells by exosomes released by gastric cancer cells, which was richer in non-metastatic gastric cancer cells than in metastatic gastric cancer cells. It indicated that gastric cancer cells disrupted the balance between epithelial cells and mesothelial cells via decreasing the downregulating miR-486-5p in the released exosomes. Our preliminary data showed that SAMD2, CDK4, and ACTR3 were significantly downregulated by miR-486-5p. Further investigations will be conducted in our future work to explore the direct target of miR-486-5p, such as dual-luciferase reporter assay, to better understand the regulatory function of miR-486-5p on EMT processing (Fig. 6).

## Conclusion

In this study, the results indicated that decreased delivery of miR-486-5p via exosomes might be responsible for the peritoneal metastasis of gastric cancer cells by promoting EMT progress.

## Data Availability

The data are available from the corresponding author on reasonable request.

## Abbreviations

Con-Exo: Exosomes from GC9811 cells
PM-Exo: Exosomes from GC9811-P cells
TEM: Transmission electron microscopy
ACTR3: Actin-related protein 3
SMAD2: Mothers against decapentaplegic homolog 2
CDK4: Cyclin-dependent kinase 4
EMT: Epithelial-mesenchymal transition
FBS: Fetal bovine serum
U6: RUN6-1
TBST: Tris-buffered saline with 0.1% Tween® 20 detergent
BSA: Bovine serum albumin
